# Quantification of sarcopenia in patients with rheumatoid arthritis by measuring the cross-sectional area of the thigh muscles with magnetic resonance imaging

**DOI:** 10.1007/s11547-023-01630-9

**Published:** 2023-04-29

**Authors:** Fausto Salaffi, Marina Carotti, Anna Claudia Poliseno, Luca Ceccarelli, Sonia Farah, Marco Di Carlo, Andrea Giovagnoni

**Affiliations:** 1 Clinica Reumatologica, Ospedale “Carlo Urbani”, Università Politecnica delle Marche, Via Aldo Moro, 25, 60035 Jesi (Ancona), Italy; 2 Dipartimento di Scienze Radiologiche, Ospedali Riuniti, Università Politecnica delle Marche, Ancona, Italy; 3 IRCCS AOUBO, Pediatric and Adult Cardio-Thoracovascular, Onchoematologic and Emergencies Radiology Unit, Bologna, Italy

**Keywords:** Rheumatoid arthritis, Sarcopenia, Magnetic resonance imaging, Handgrip strength, Physical performance

## Abstract

**Purpose:**

To determine the utility of cross-sectional area (CSA) measurements on magnetic resonance imaging (MRI), at the level of the thigh muscles, to estimate muscle mass in discriminating rheumatoid arthritis (RA) patients with sarcopenia from those without.

**Materials and methods:**

Consecutive female RA patients were enrolled for this cross-sectional study. Patients were assessed for disease activity, radiological damage, handgrip strength, physical performance and for the presence of sarcopenia, identified in accordance with the EWGSOP2 criteria. A 1.5 T MRI machine was used to scan the thigh muscles. A dimensional region growth algorithm (Horos™) was used to segment the muscles CSAs (in cm^2^) on MR images located 25 cm above the knee joint (MRI-CSA-25). The MRI-CSA-25 was obtained by summing the CSAs of the individual muscles. MRI-CSA-25 was correlated (Pearson's r) with the other variables, and its optimal cut-off point (Youden index) for sarcopenia diagnosis was identified in relation to the EWGSOP2 criteria.

**Results:**

32 RA female patients were studied, 34.4% diagnosed as sarcopenic**.** The mean MRI-CSA-25 was 151.00 cm^2^ for patient with sarcopenia, 275.57 cm^2^ for patient without sarcopenia (*p* < 0.001). MRI-CSA-25 correlated significantly with measures of physical performance, and disease activity, but not with radiological damage or age. The MRI-CSA-25 optimal cut-off point in discriminating sarcopenic patients was identified at 182.00 cm^2^ (AUC-ROC = 0.894).

**Conclusion:**

MRI-CSA-25 can differentiate sarcopenic versus non-sarcopenic RA patients, representing an imaging biomarker of this condition.

**Supplementary Information:**

The online version contains supplementary material available at 10.1007/s11547-023-01630-9.

## Introduction

Sarcopenia is a prevalent extra-articular manifestation in patients suffering from rheumatoid arthritis (RA) [1]. It is difficult to recognise sarcopenia in the clinical setting as there are no methods for determining muscle mass that are widely applicable in everyday practice. The European Working Group on Sarcopenia in Older People (EWGSOP2) currently defines sarcopenia as a 'syndrome characterised by progressive and generalised loss and changes in skeletal muscle mass and strength' [2]. Rheumatoid sarcopenia is a multifactorial condition, including hormonal, inflammatory, and physical inactivity determinants. In the course of RA, protein catabolism under the drive of pro-inflammatory cytokines such as tumor necrosis factor and interleukin-6 may be particularly pronounced [3].

Although specific diagnostic criteria for sarcopenia are still being developed, imaging techniques are central to measuring the quantity or estimating the quality of muscle mass [4-6].

Dual-energy X-ray Absorptiometry (DXA) assessment of body composition has been used as an endpoint in clinical trials involving different populations with a wide range of diseases for over a decade [7, 8]. The ability to rapidly assess whole-body composition is the main advantage of DXA over other methods. In accordance with the EWGSOP2 consensus, DXA is the gold standard technique for confirming the diagnosis of sarcopenia [2].

Sonography is known for its accessibility, low cost ease of use and feasibility [9-11]. Ultrasound (US) imaging can be heavily affected by variables intrinsic to the technique itself, such as reproducibility of transducer placement and compression, by patient-related variables, such as body habitus, and by muscle-related variables, such as contraction or resting state [12]. Despite these obstacles, parameters such as cross-sectional area (CSA) and volume, measured using panoramic images, offer inter- and intra-observer reliability comparable to that of magnetic resonance imaging [MRI] [13].

Computed tomography (CT) images are frequently used as diagnostic means for sarcopenia, and muscles measurements are employed as biomarker of sarcopenia. CT has a major disadvantage in terms of radiation exposure, and recommending CT execution with the only purpose of screening for sarcopenia is currently inapplicable [4].

MRI could be a promising option for the evaluation of sarcopenia. On the other hand, the widespread use of MRI for the evaluation of sarcopenia is limited by the lack of standardisation of protocols, high costs and difficult post-processing. MRI allows the measurement of CSA and volume of a muscle and the visualization of its morphological features and their distribution. Quality deterioration of the muscle, characterised by the presence of adipose tissue (defined as myosteatosis) and of fibrous connective tissue (defined as myofibrosis), is best diagnosed with MRI [14, 15]. However, a correct segmentation of the muscle to define the regions of interest is necessary for a definition of the typical changes in sarcopenia [16]. Manual contouring of the muscle from surrounding tissues, performed on T1-weighted images with an adaptive threshold, has been proposed as an automatic method to segment the quadriceps femoris [17].

The aim of this study was to determine the utility of MRI-CSA measurements at the level of the thigh (located 25 cm above the knee joint) (MRI-CSA-25) to estimate muscle mass in discriminating RA patients with sarcopenia from those without, and to study the relationships between MRI-CSA-25 and the measures of physical performance and indices of disease activity.

## Methods

### Design and study population

The study enrolled consecutive female RA patients. RA diagnosis was formulated in accordance to the 2010 criteria of the American College of Rheumatology/European League Against Rheumatism (ACR/EULAR) [18]. RA patients have been included at the Rheumatology Clinic, Università Politecnica delle Marche, “Carlo Urbani” Hospital, Jesi (Ancona), between June 2021 and August 2022.

Patients were included regardless of disease activity status and without contraindications to MRI. Patients with co-existing neurological, muscular, cardiovascular, pulmonary, renal, oncological diseases, chronic infections, or who were taking medication that could influence the study variables, were excluded.

### Demographic data, anthropometric variables, comorbidities and laboratory investigations

Age, level of education, disease duration (defined as time since diagnosis), body mass index (BMI), and current treatment [glucocorticoids, conventional synthetic disease modifying anti-rheumatic drugs (csDMARDs) and/or biologic DMARDs (bDMARDs)], were variables included in the study. Comorbidities burden was estimated with the modified Rheumatic Disease Comorbidity Index (mRDCI) [19].

For each patients were registered the erythrocyte sedimentation rate (ESR) in mm/h, the C-reactive protein (CRP) in mg/dl, the presence of IgM-rheumatoid factor (RF) and of anti–citrullinated protein antibodies (ACPA).

### Composite disease activity indices

Disease activity was assessed using the Simplified Disease Activity Index (SDAI). SDAI is based on a linear sum of five variables: counts for swollen and tender joints on 28 joints (SJC and TJC, respectively), patient and physician assessments of disease activity (PhGA and PaGA, respectively) on 0–10 numerical rating scales (NRS), and CRP (in mg/dl). SDAI ranges from 0 to 86. The cut-off values distinguishing remission (REM), low disease activity (LDA), and moderate disease activity (MDA) are 3.3, 11 and 26, respectively [20].

### Radiographic scoring

Radiographs of hands, wrists and feet were used to estimate the radiographic damage and scored according to the Simple Erosion Narrowing Score (SENS) [21] by two readers (FS and MC) with experience with this method. SENS accurately reflects radiographic development assessing the same joints included in Sharp-van der Heijde score for the presence of erosion (if present, scored 1 for each joint) and joint space narrowing (if present, scored 1 for each joint). The SENS ranges from 0 to 86 [22].

### Diagnosis of sarcopenia

Sarcopenia was diagnosed according to the EWGSOP2 operational definition [2]. EWGSOP2 definition applies low muscle strength as the primary variable for sarcopenia diagnosis which is “probable” when low muscle strength is documented. The diagnosis is established by the presence of low muscle quantity or low muscle quality. If low muscle strength, low muscle quantity/quality, and low physical performance are coexisting, sarcopenia is defined severe. For the purposes of this study, the diagnosis of sarcopenia was confirmed by US detection of low muscle quality, arbitrarily defined by the presence of grades 2 and 3 of increased echogenicity of the rectus femoris and vastus intermedius muscles on a semiquantitative scale [10], as described in detail below.

EWGSOP2 recommends the SARC-F as screening tool. SARC-F is a self-reported 5-item questionnaire that estimate the sarcopenia risk according to the patient’s perception of limitations in different daily life domains (strength, walking ability, rising from a chair, stair climbing and falls) [23]. SARC-F has been used as a screening measure in the diagnostic flow-chart but has not played a role in confirming the diagnosis of sarcopenia.

### Handgrip strength assessment

The handgrip strength (HGs) was measured using a cylindrical-shaped grip device with five force sensors connected to a microprocessor [24]. HGs was measured twice in the dominant hand, considering the mean of the two results for the analyses, and expressed in kilograms. HGs was estimated with patients sitting in a standardised position, with the elbows flexed at a 90-degree angle and the forearm in a neutral position [25]. The cut-off used for low HGs was < 16 kg, as indicated by EWGSOP2 for women [2].

### Physical performance

Physical performance is a multidimensional concept involving functions of the whole body and related to locomotion. It refers to the integrated action of muscles, central and peripheral nerve functions such as balance, and can be measured with the Short Physical Performance Battery (SPPB). SPPB includes three evaluations: 1) repeated chair stands; 2) balance tests (side-by-side, semi tandem and tandem balance tests); 3) an eight-foot walk test. Each assessment is graded on a four-point scale, with the results of the three tests added together to provide a total score ranging from 0 (worst result) to 12 (best result) [26]. According to EWGSOP2, in this study low performance was identified for SPPB ≤ 8 [2].

### Ultrasound assessment of thigh muscle

**Fig. 1:**
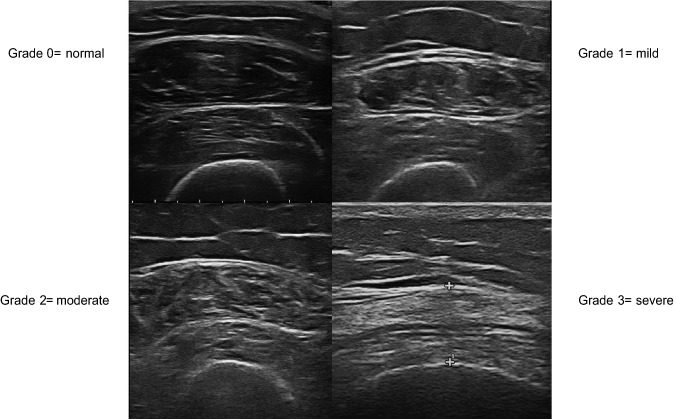
Illustrative reference images for semi-quantitative ultrasound evaluation of the rectus femoris and vastus intermedius muscles

All the US examinations were carried out using a MyLab ClassC (Esaote SpA) provided with a broadband linear probe (frequency range 4–13 MHz). The US examinations were performed on the dominant lower limb. A rheumatologist trained in musculoskeletal US (MDC, with 10 years of experience in musculoskeletal US) carried out the US examinations on the patients adopting a protocol described previously [11]. US was conducted with patients lying supine and with the lower limbs extended, relaxed and avoiding muscle contraction and extrotation of the hips. The focus of US assessment was at the midpoint between the upper pole of the patella and the anterior superior iliac spine (ASIS). The US was conducted with transverse scans, with an adequate amount of gel to avoid compression of the underlying tissues. Specifically, with the US beam perpendicular to the long axis of the femur, the probe was moved slightly (laterally or medially to the previously identified skin landmark) to optimally visualize the belly of the rectus femoris muscle, acquiring images with the rectus femoris muscle in the center of the screen and the vastus intermedius muscle below it. On the US images obtained, rheumatologists (FS and MDC) were asked to consensually score muscle echogenicity of rectus femoris and vastus intermedius muscles according to a visual semi-quantitative scale, recently developed, grading muscle echogenicity from 0 to 3: 0 = normal (normal hypoechoic muscle), 1 = mild (increased echogenicity regarding less than one-third of muscle tissue), 2 = moderate (increased echogenicity in more than one-third but less than two-thirds of muscle tissue), and 3 = severe (increased echogenicity in more than two-thirds of muscle tissue) (Fig. 1) [10].

### MRI examination and images analysis

MRI of the thighs was performed using a 1.5 T scanner with a 16-channel “dStream torso coil” coil (Achieva Philips Medical Systems, Best, the Netherlands) (Supplementary Table 1). Examinations were performed in supine position. All the MRI examinations were assessed independently by two musculoskeletal radiologists (MC and ACP, with an expertise of 5 and 20 years in interpreting MRI images, respectively) blinded to clinical, US and laboratory data. To establish intra-rater reliability, the two radiologists performed the images analysis two week later.

**Fig. 2:**
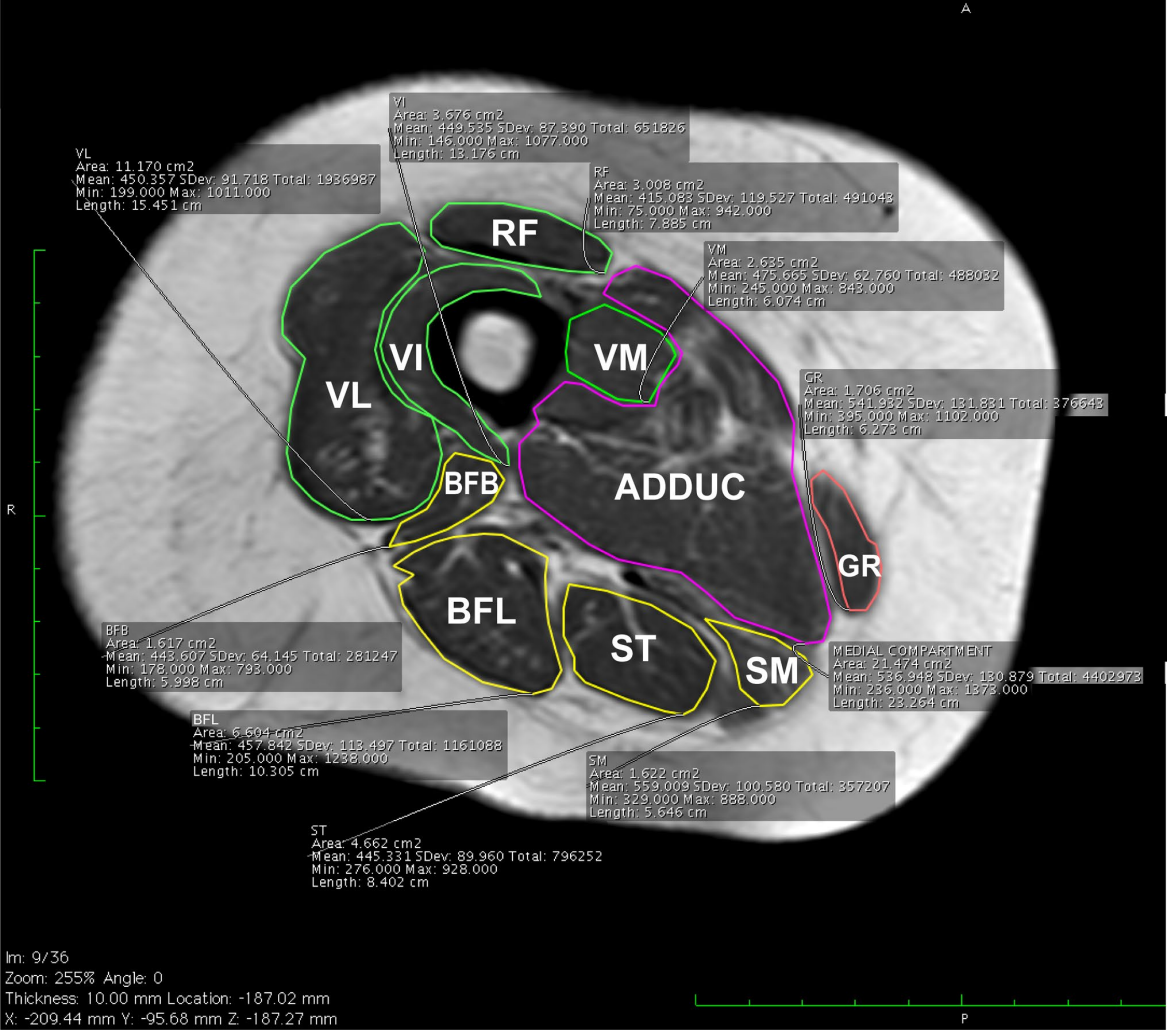
Illustrative magnetic resonance image of a segmented muscle region of the thigh at a 25 cm above the knee joint (MRI-CSA-25) of a 50-year-old female patient, acquired as axial T1-weighted gradient-echo. Group muscles are color-coded: rectus femoris (RF), vastus lateralis (VL), vastus intermedius (VI), and vastus medialis (VM), the four muscles composing the hamstrings muscles (HAMST) [biceps femoris short head (BFB), biceps femoris long head (BFL), semitendinosus (ST) and semimembranosus (SM], and adductors (ADDUC) [adductor longus, brevis, magnus, pectineus, sartorius and gracilis (GR)]. Because the delineations between adductors muscles were not so obvious at proximal part, all adductors’ muscles were segmented together, except for the gracilis which was easily recognizable. Note the exclusion of non-muscular elements (blood vessels, nerves) in the manual segmentation

Images analysis was carried out using Horos™, an open-source medical image viewer to segment the quadriceps muscle CSA (expressed in cm^2^). Horos™ is available under the GNU Lesser General Public License, Version 3 (LGPL-3.0). Muscles have been segmented with the “closed polygon” function that allows automatic measurement of CSA. With one click on the image, points can be placed and used to create the polygon. After placing the third point, a line is drawn connecting the last placed point to the first. This tool is useful for delineating and finding the area (and volume) of structures. In segmetation, the four components of the quadriceps muscle (rectus femoris, vastus lateralis, vastus intermedius, and vastus medialis), the four components of the hamstrings muscles (biceps femoris short head, biceps femoris long head, semitendinosus and semimembranosus), and the adductors (adductor longus, brevis, magnus and pectineus, the sartorius and gracilis) have been included. Since identification of the individual components of the adductor muscles is not straightforward in the region chosen for segmentation, they were all considered together with the exception of the gracilis muscle, which is well recognizable (Fig. 2) [27]. Adipose tissue, femoral bone, blood vessels and nerves were excluded as far as possible from the segmented muscle regions. The muscle CSAs selected for analyses were located in the thigh, 25 cm above the knee joint (CSA-25) [28]. MRI-CSA-25 estimation was performed on a single scan, also to simplify image acquisition and processing [27, 29].

### Statistical analysis

The analyses were carried out with MedCalc Statistical Software, version 19.0 (Ostend, Belgium). Categorical variables were reported in terms of descriptive statistics using numbers and percentages, while continuous variables were reported using mean, standard deviation (SD) and median. Percentage differences between groups were analyzed using Chi-square test or Fisher's exact test. The intra-class correlation coefficient (ICC) was used to evaluate the intra-observer reliability for the repeated MRI-CSA-25 measurements, interpreting an ICC > 0.80 as indicative of “almost perfect” agreement [30]. The association between the MRI-CSA-25 and the other variables (US score, SPPB, SARC-F, HGs, age, mRDCI, and SDAI) was investigated with the Pearson correlation analysis, interpreting the strength of correlations: low = 0.26–0.49, moderate = 0.50–0.69, high = 0.70–0.89, and very high = 0.90–1.00. The accuracy of MRI-CSA-25 in detecting sarcopenia was compared to EWGSOP2 using the receiver operating characteristic (ROC) curve analysis, using the Youden index as optimal cut-off point.

## Results

### Demographic and disease characteristics

Thirty-two female patients, with a mean age of 71.3 years (range, 39–81 years), a mean disease duration of 8.6 years (range, 3–21 years) have been included in the study. ACPA were detectable in 65.6% of patients and RF in 71.8%. All the following results are expressed as mean ± standard deviation. BMI was 23.5 (± 3.3) kg/m^2^, while regarding disease severity, the SDAI was 25.8 (± 9.8) and the SENS was 31.1 (± 20.7). The most frequent comorbidities were cardiovascular (31.2%), followed by respiratory (18.7%) and metabolic (15.6%) disorders (Table 1).

**Table 1:** Summary statistic of the patients investigated

	Mean	SD
Age (years)	71.34	10.98
BMI (kg/m^2^)	23.47	3.28
Disease duration (years)	8.62	7.02
mRDCI	2.09	1.27
SDAI	25.76	14.70
SENS	31.09	20.67
SARC-F	4.46	2.14
Handgrip strength	26.20	11.68
US score	1.65	0.82
SPPB	6.12	2.18
MRI-CSA-25	232.75	94.58

*SD* = standard deviation; *BMI* = body mass index; *mRDCI* = modified Rheumatic Disease Comorbidity Index; *SDAI* = Simplified Disease Activity Index; *SENS* = Simple Erosion Narrowing Score; *SARC-F* = Screen Questionnaire for Sarcopenia; *US* = ultrasound; *SPPB* = Short Physical Performance Battery; *MRI* = magnetic resonance imaging; *CSA-25* = cross-sectional area at a 25 cm above the knee joint

The 100% of patients was receiving a bDMARD, respectively, 10 (31.2%) etanercept, 7 (21.8%) adalimumab, 6 (18.8%) abatacept, 5 (15.6%) golimumab, and 4 (12.5%) tocilizumab. Most of the patients were on their first bDMARD. The 63% of the patients received also a csDMARD, usually methotrexate (80.0%). Fifteen patients (46.8%) were assuming oral corticosteroids with a mean prednisone (or equivalent) dose of 3.6 mg/day (range 2.5–16), and 18 (56.2%) were prescribed non-steroidal anti-inflammatory drugs (NSAIDs) on demand.

**Fig. 3:**
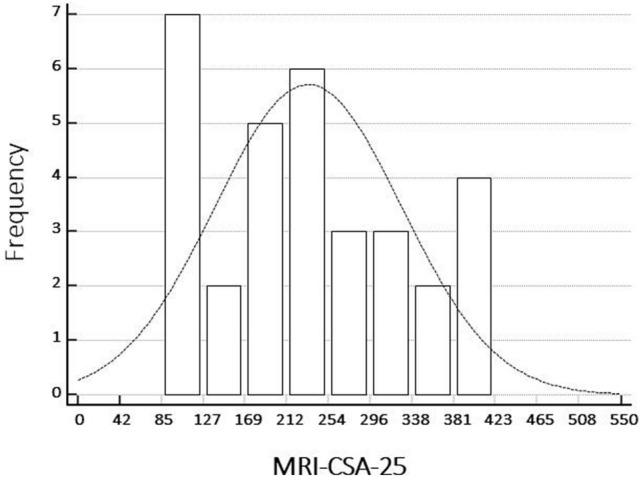
Distribution of the values of MRI-CSA-25 in segmented thigh muscles of the whole cohort

The SARC-F was 4.4 (± 2.1), the HGs was 26.2 (± 11.7), while the US semiquantitative score was 1.6 (± 0.8). Eleven (34.4%) of the 32 patients were diagnosed as sarcopenic according to EWGSOP2 operational definition. The MRI-CSA-25 for the entire group was 232.75 (± 94.58) cm^2^, 151.00 (± 62.85) cm^2^ for patient with sarcopenia (11 patients) versus 275.57 (± 79.30) cm^2^ for patient without sarcopenia (*p* < 0.001) (Table 2). The descriptive analysis of the values of MRI-CSA-25 detected in MRI is reported in Fig. 3, where a parametric distribution is highlighted.

**Table 2:** Summary statistics and differences in RA patients without and with sarcopenia

	Patients without sarcopenia (n, 21)		Patients with sarcopenia (n, 11)		Significance
	Mean	SD	Mean	SD	*p*
Age (years)	71.61	12.29	70.81	8.45	ns
BMI (kg/m^2^)	23.82	3.08	22.79	3.69	ns
Disease duration (years)	8.09	7.64	9.63	5.85	ns
mRDCI	1.76	1.17	2.72	1.27	0.040
SDAI	16.53	7.00	43.39	7.18	< 0.001
SENS	32.57	20.63	28.27	21.46	ns
SARC-F	3.04	0.49	7.18	1.16	< 0.001
Handgrip strength	33.59	6.16	12.09	3.66	< 0.001
SPPB	7.47	1.12	3.54	1.03	< 0.001
MRI-CSA-25	275.57	79.30	151.00	62.85	< 0.001
US score	1.33	0.65	2.27	0.78	< 0.001

*SD* = standard deviation; *BMI* = body mass index; *mRDCI* = modified Rheumatic Disease Comorbidity Index; *SDAI* = Simplified Disease Activity Index; *SENS* = Simple Erosion Narrowing Score; *SARC-F* = Screen Questionnaire for Sarcopenia; SPPB = Short Physical Performance Battery; *MRI* = magnetic resonance imaging; *CSA-25* = cross-sectional area at a 25 cm above the knee joint; *US* = ultrasound

The intra-observer agreement in assessing MRI-CSA-25 between the two operators was 0.991, with a standard error of 0.053, and a 95% confidence interval (CI) 0.881–0.998.

### Correlation between MRI-CSA-25 and other variables

MRI-CSA-25 correlated significantly with the other sarcopenia and performance measures, HGs (r = 0.714, *p* < 0.0001), SARC-F (r = -0.550, *p* = 0.0011), SPPB (r = 0.542; *p* = 0.0013), and US score (r = − 0.692, *p* < 0.0001), respectively. There was also a significant correlation between MRI-CSA-25 and disease activity assessed with SDAI (r = − 0.616, *p* = 0.0002), while there was no correlation with radiographic damage estimated with SENS (r = − 0.022, *p* = 0.904) (Table 3).

**Table 3:** Correlation table (Pearson’s r) among the variables studied

	MRI-CSA-25	US score	SPPB	SARC-F	Handgrip strength	Age	mRDCI	SDAI
US score	− 0.692^ < 0.0001*							
SPPB	0.542^ 0.001*	− 0.458^ 0.008*						
SARC-F	− 0.550^ 0.001	0.440^ 0.011*	− 0.849^ < 0.0001*					
Handgrip strength	0.714^ < 0.0001*	− 0.566^ 0.0007*	0.790^ < 0.0001*	− 0.830^ < 0.0001*				
Age	− 0.066^ 0.7199*	− 0.029^ 0.874*	− 0.150^ 0.412*	− 0.033^ 0.857*	0.025^ 0.890*			
mRDCI	− 0.210^ 0.247*	0.184^ 0.313*	− 0.363^ 0.041*	0.313^ 0.080*	− 0.340^ 0.056*	0.021^ 0.910*		
SDAI	− 0.616^ 0.0002*	0.504^ 0.003*	− 0.736^ < 0.0001*	0.843^ < 0.0001*	− 0.883^ < 0.0001*	− 0.119^ 0.515*	0.466^ 0.007*	
SENS	− 0.022^ 0.904*	0.117^ 0.523*	− 0.029^ 0.875*	− 0.030^ 0.869*	0.118^ 0.520*	0.382^ 0.030*	0.392^ 0.026*	0.004^ 0.983*

*MRI* = magnetic resonance imaging; *CSA-25* = cross-sectional area at a 25 cm above the knee joint; *US* = ultrasound; *SPPB* = Short Physical Performance Battery; *SARC-F* = Screen Questionnaire for Sarcopenia; *mRDCI* = modified Rheumatic Disease Comorbidity Index; *SDAI* = Simplified Disease Activity Index; *SENS* = Simple Erosion Narrowing Score; ^ = r values; * = *p* values

### ROC curve analysis for the performance of the MRI-CSA-25

**Fig. 4:**
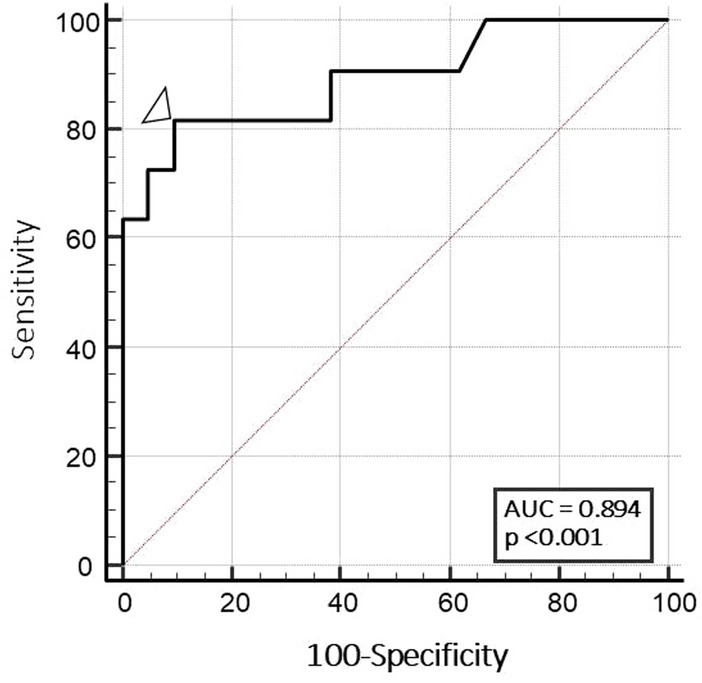
Receiver operating characteristic (ROC) curve analysis for the performance of the MRI-CSA-25 in discriminating RA patients with sarcopenia from those without. The triangle shows the optimal cut-off point (182.00 cm^2^) corresponding to the highest sensitivity (81.8%) and specificity (90.5%) combination. Area under the ROC curve is 0.894 (standard error 0.066 and 95% CI 0.765–1.000)

Compared to diagnosis of sarcopenia according to the EWGSOP2 operational definition, MRI-CSA-25 showed a significant performance (*p* < 0.001) in discriminating sarcopenic versus non-sarcopenic patients, with an AUC-ROC of 0.894 (Fig. 4) and an optimal cut-off point of 182.00 cm^2^ (sensitivity 81.82% [95% CIs 48.2–97.7], specificity 90.51% [95% CIs 69.6–98.8], positive likelihood ratio 8.59 [95% CIs 2.2–33.1]) (Supplementary Table 2).

## Discussion

To our knowledge, this is the first study applying MRI in the assessment of midthigh muscle mass in RA patients to assess sarcopenia.

Awareness of sarcopenia in immune-mediated rheumatic diseases (IMRDs) is crucial for both the radiologist and the rheumatologist: the former is responsible for diagnosis through imaging techniques, the latter for proper clinical management. RA predisposes to sarcopenia with an odd ratio of 1.83 [31], and systemic inflammation caused by pro-inflammatory cytokines is one of its major contributor [32]. Cross-sectional investigations found that the overall prevalence of sarcopenia in RA patients was significantly greater than in controls [32-38]. The lack of a universally accepted definition of sarcopenia accounts for rather different prevalences in different case series. A 2017 study, in which the diagnosis of sarcopenia identified solely by DXA parameters, documented a prevalence of the condition in 39.8% of RA patients [39]. In contrast, a more recent study assessing the presence of sarcopenia in RA, again performing body composition assessment by DXA but including it in the operational definition proposed by EWGSOP2, found a prevalence of sarcopenia of 4.5% of patients compared with 0.4% of healthy controls [40]. The prevalence of sarcopenia found in the present study, albeit DXA was not used, is within this range.

Although DXA is still considered the reference technique in the assessment of lean mass, over the past years CT and MRI are emerging as reference methods in the assessment of specific muscle groups. MRI, exploiting the contrast between fat and water, is the optimal technique for evaluating muscle and adipose tissue. Skeletal muscle composition analyses on MRI have generally been performed with purely manual segmentation into a region of interest (ROI). This type of analysis is associated with high inter-operator variability and is time-consuming [41, 42]. To overcome these limitations, automatic methods that use thresholds on MRI images to assess CSA have gained acceptance over the years. The CSA of the muscles of the thigh is the one most frequently measured in studies and considered an important clinical indicator. Measurement of the CSA of a muscle using an automatic method is relatively easy to perform and is reproducible, two features that support its clinical applications. A recent study found a strong correlation between manual and automatic measurement, with the latter method also being 70% faster [43]. Excellent intra-observer concordance was also found in the present study, mirroring that of the data in the literature [44, 45].

There are several approaches to evaluate CSA of the muscles of the thigh, with a lack of true standardisation for this measurement, particularly with regard to where to take the measurement [46, 47]. A study that compared CSA at multiple levels of the quadriceps femoris versus volumetric quantification suggests that CSA-25 is the most appropriate [28].

The present study has several limitations. The first concern the low sample size and the absence of a control group in which to compare the performance of MRI-CSA-25. In the second instance, it should be mentioned that sarcopenia was confirmed using US, whereas EWGSOP2 indicates more validated techniques (such as DXA or CT) as the reference diagnostic method. However, US of the anterior thigh has also already been successfully used by other research groups for the diagnosis of sarcopenia in patients with RA [48]. Thirdly, for practical reasons, the measurement of the CSA was limited to one thigh level, without further CSA measurements and without volumetric reconstructions. In addition the adductor muscles were considered together, including the fascia and possibly a small amount of adipose tissue interposed between the muscle bellies. This may have introduced a systematic error that could, however, have influenced the CSA values in a similar way, without therefore having a major impact on the correlation analysis.

In conclusion, while considering the limitations of the study, it can be affirmed that MRI-CSA-25 at thigh level was able to distinguish RA patients with sarcopenia from those without. Furthermore, a strong link was found between this measurement and physical function and disease activity. Accurate assessment, through imaging techniques, of lean mass in major locomotor muscle groups is important to support the diagnosis of a complex multifactorial condition such as sarcopenia. Future automated techniques could improve the reliability and accuracy of MRI assessment.

## Supplementary Information

Below is the link to the electronic supplementary material.

Supplementary file 1 (DOCX 33 KB)
