# Supplementary material for: Quantification of sarcopenia in patients with rheumatoid arthritis by measuring the cross-sectional area of the thigh muscles with magnetic resonance imaging
Source: Radiol Med. 2023 Apr 29;128(5):578–87. doi: 10.1007/s11547-023-01630-9 (PMC10182126; doi:10.1007/s11547-023-01630-9)
Supplement: Supplementary file 1 — (DOCX 33 KB) [file 11547_2023_1630_MOESM1_ESM.docx]

**Supplementary Table 1**. Magnetic resonance imaging protocol.

| Sequence plane | Parameters |
| --- | --- |
| T2 TSE axial | FOV = 300x455, Matrix = 332x394, slice thickness = 10 mm, gap = 2 mm, TR = 3000-6666 msec, TE = 100 msec, flip angle:90° |
| T1 TSE axial | FOV = 300x455, Matrix = 300x392, slice thickness = 10 mm, gap = 2 mm, TR = 455 msec, TE = 8 msec, flip angle:90° |
| STIR axial | FOV =250x454, Matrix = 196x271, slice thickness = 10 mm, gap = 2 mm, TR = 2500-6500 msec, TE = 55 msec, TI = 150 msec |
| T1 TSE coronal | FOV = 455x403, Matrix = 456x362, slice thickness = 10 mm, gap = 2 mm, TR = 510 msec, TE = 10 msec, flip angle:90° |
| STIR coronal | FOV = 455x398, Matrix = 384x255, slice thickness = 10 mm, gap = 2 mm, TR = 2500-6500 msec, TE = 50 msec, TI = 150 msec |

**Supplementary Table 2.** Criterion values and coordinates of the receiver operating characteristic curve in discriminating rheumatoid arthritis patients with and without sarcopenia.

| Criterion | Sensitivity | 95% CI | Specificity | 95% CI | +LR | 95% CI | -LR | 95% CI |
| --- | --- | --- | --- | --- | --- | --- | --- | --- |
| <103 | 0.00 | 0.0 - 28.5 | 100.00 | 83.9 - 100.0 |  |  | 1.00 | 1.0 - 1.0 |
| ≤122 | 63.64 | 30.8 - 89.1 | 100.00 | 83.9 - 100.0 |  |  | 0.36 | 0.2 - 0.8 |
| ≤132 | 63.64 | 30.8 - 89.1 | 95.24 | 76.2 - 99.9 | 13.36 | 1.9 - 95.3 | 0.38 | 0.2 - 0.8 |
| ≤155 | 72.73 | 39.0 - 94.0 | 95.24 | 76.2 - 99.9 | 15.27 | 2.2 - 107.0 | 0.29 | 0.1 - 0.8 |
| ≤175 | 72.73 | 39.0 - 94.0 | 90.48 | 69.6 - 98.8 | 7.64 | 1.9 - 30.0 | 0.30 | 0.1 - 0.8 |
| ≤182* | 81.82 | 48.2 - 97.7 | 90.51 | 69.6 - 98.8 | 8.59 | 2.2 - 33.1 | 0.20 | 0.06 - 0.7 |
| ≤232 | 81.82 | 48.2 - 97.7 | 61.90 | 38.4 - 81.9 | 2.15 | 1.2 - 4.0 | 0.29 | 0.08 - 1.1 |
| ≤233 | 90.91 | 58.7 - 99.8 | 61.90 | 38.4 - 81.9 | 2.39 | 1.3 - 4.2 | 0.15 | 0.02 - 1.0 |
| ≤294 | 90.91 | 58.7 - 99.8 | 38.10 | 18.1 - 61.6 | 1.47 | 1.0 - 2.2 | 0.24 | 0.03 - 1.7 |
| ≤298 | 100.00 | 71.5 - 100.0 | 33.33 | 14.6 - 57.0 | 1.50 | 1.1 - 2.0 | 0.00 |  |
| ≤409 | 100.00 | 71.5 - 100.0 | 0.00 | 0.0 - 16.1 | 1.00 | 1.0 - 1.0 |  |  |

Abbreviations and legends: CI = confidence interval; +LR = positive likelihood ratio; -LR = negative likelihood ratio; * = optimal cut-off point.
